# Prevalence, quantification, and household-level risk factors associated with *Salmonella* spp. infection in chickens in Boussouma commune, Burkina Faso

**DOI:** 10.1186/s12866-026-04895-y

**Published:** 2026-03-04

**Authors:** Wendenso Patrick Bertrand Tiendrébéogo, Assèta Kagambèga, Arshnee Moodley, Linnet Ochieng, Tho Abou Da, Rasmané Tao, Abdoul Kader Ilboudo, Brice Ouedraogo, Guy Ilboudo, Nicolas Barro, Michel Dione

**Affiliations:** 1 Laboratory of Molecular Biology, Epidemiology and Surveillance of Foodborne Bacteria and Viruses, Department of Biochemistry-Microbiology, Doctoral School of Science and Technology, Joseph KI-ZERBO University, Ouagadougou, 03 BP 7021 Burkina Faso; 2 École Normale Supérieure, Ouagadougou, Burkina Faso; 3 International Livestock Research Institute, HEALTH program, Nairobi, Kenya; 4 Department of Veterinary and Animal Science, University of Copenhagen, Frederiksberg C, Denmark; 5 International Livestock Research Institute, Ouagadougou, Burkina Faso

**Keywords:** Biosecurity, One Health, *Salmonella* spp., Village chicken, Zoonotic transmission

## Abstract

**Background:**

Village chicken production is central to rural livelihoods across sub-Saharan Africa. However, the poor biosecurity and hygiene observed in the chicken flocks, coupled with the limited access of farmers to veterinary services, provide favorable conditions for the persistence and transmission of zoonotic pathogens such as *Salmonella* spp. This study aimed to estimate the prevalence of *Salmonella* in village chicken flocks; quantify fecal bacterial loads; and identify household – and flock-level risk factors associated with *Salmonella* spp. infection in the Boussouma commune in rural Burkina Faso.

**Methods:**

A cross-sectional study was conducted in 73 poultry-keeping households. Cecal contents were collected from 292 indigenous chickens following humane euthanasia. Laboratory analysis was carried out using the ISO 6579:2012 standard methods. Quantification of *Salmonella* spp. In fecal matters, it was done using the Most Probable Number (MPN) method. Structured household interviews captured data on poultry management, hygiene practices, flock characteristics, and household demographics. Multivariate logistic and linear regression models were used to assess associations between household- and flock-level practices with *Salmonella* spp. presence and bacterial load.

**Results:**

The animal-level prevalence of *Salmonella* spp. in chicken faeces was 57.2% (95% CI: 51.5–62.9). *Salmonella* spp. loads in faeces ranged from 0.03 to 10.99 MPN/g (mean = 0.63 MPN/g). In multivariate logistic regression, lack of access to veterinary care (OR = 3.77; *p* = 0.001), on-site accumulation of poultry manure (OR = 5.61; *p* = 0.011), and burial of dead chickens within the household compound (OR = 1.92; *p* = 0.024) were associated with increased odds of infection. Protective factors included improved access to water (OR = 0.46; *p* = 0.020) and removal of manure from the household environment (OR = 0.44; *p* = 0.013). Chickens from male-headed households had lower odds of infection (OR = 0.22; *p* = 0.029). Higher *Salmonella* spp. loads were associated with poor hygiene, limited water access, and lack of veterinary care.

**Conclusion:**

The findings highlight critical, context-specific points of intervention for reducing zoonotic transmission risks at the animal-household interface. Targeted community-level hygiene promotion, improved water access, safer carcass and manure management, and strengthening of village-level veterinary services are essential to mitigate public health risks linked to village poultry production.

**Supplementary Information:**

The online version contains supplementary material available at 10.1186/s12866-026-04895-y.

## Introduction

Poultry production is a key source of livelihood, income, and food security for millions of households across Sub-Saharan Africa, including Burkina Faso, where more than 1.6 million small-scale producers are involved in the sector [1, 2]. However, when poultry are raised under poor husbandry and hygiene conditions, flocks are highly exposed to several pathogens, including zoonotic pathogens such as *Salmonella* spp. Therefore, poultry production systems in low-resource settings face major public health challenges linked to the circulation of foodborne and environmentally transmitted pathogens [3]. Recent studies continue to highlight the persistence of *Salmonella* spp. in smallholder poultry systems and the difficulty of controlling transmission under extensive production conditions [4, 5].

Globally, *Salmonella* spp. infections remain among the leading causes of foodborne disease, responsible for hundreds of millions of diarrheal cases annually [3, 6]. In high-income settings, human salmonellosis is commonly associated with the consumption of contaminated raw or undercooked poultry meat or eggs [7]. In contrast, in low-resource rural settings, transmission pathways are often broader and include poor household hygiene conditions, lack of biosecurity, and unsafe manure management, and close and continuous contact between humans and poultry [8, 9]. In such settings, environmental contamination of the household compound, water sources, food preparation areas, and children’s play areas by poultry faeces represents a critical and often underestimated route of exposure to *Salmonella* spp.

The commune of Boussouma is a rural area with a high number of internally displaced persons resulting from ongoing insecurity, where village poultry production plays a crucial role in household income diversification, food security, and livelihood resilience [10]. Poultry production systems in this area are predominantly extensive, characterized by free-ranging birds, minimal housing, limited veterinary supervision, and low adoption of biosecurity measures, conditions that favor the maintenance and spread of zoonotic pathogens such as *Salmonella* spp. Similar structural and management-related vulnerabilities have been reported in recent studies of village poultry systems, highlighting consistent risk patterns across different rural contexts [4, 5].

Unlike in high-income countries, poultry products are not consumed daily: chickens are primarily kept for sale, ceremonies, or breeding, while eggs are mainly used for hatching [9, 10]. Consequently, human exposure to *Salmonella* spp. in rural households is more likely to occur through environmental contamination of water, food, and household surfaces with poultry excreta than through the direct consumption of contaminated meat or eggs [11, 12].

Despite the public health relevance of village poultry, data on the burden of *Salmonella* spp. infection and the associated management-related risk factors in rural poultry systems in Burkina Faso remain limited. Moreover, few studies have quantified fecal *Salmonella* spp. loads in village chickens, despite their importance for understanding environmental contamination pressure and transmission potential. Addressing these gaps is particularly relevant in light of recent calls for context-specific epidemiological data to inform locally adapted control strategies in smallholder poultry systems [13].

This study, conducted in the municipality of Boussouma, sought to (1) determine the prevalence of *Salmonella* spp. in village chickens, (2) determine bacterial loads, and (3) identify household- and flock-level risk factors associated with *Salmonella* spp. infection.

## Materials and methods

### Study area

**Fig. 1:**
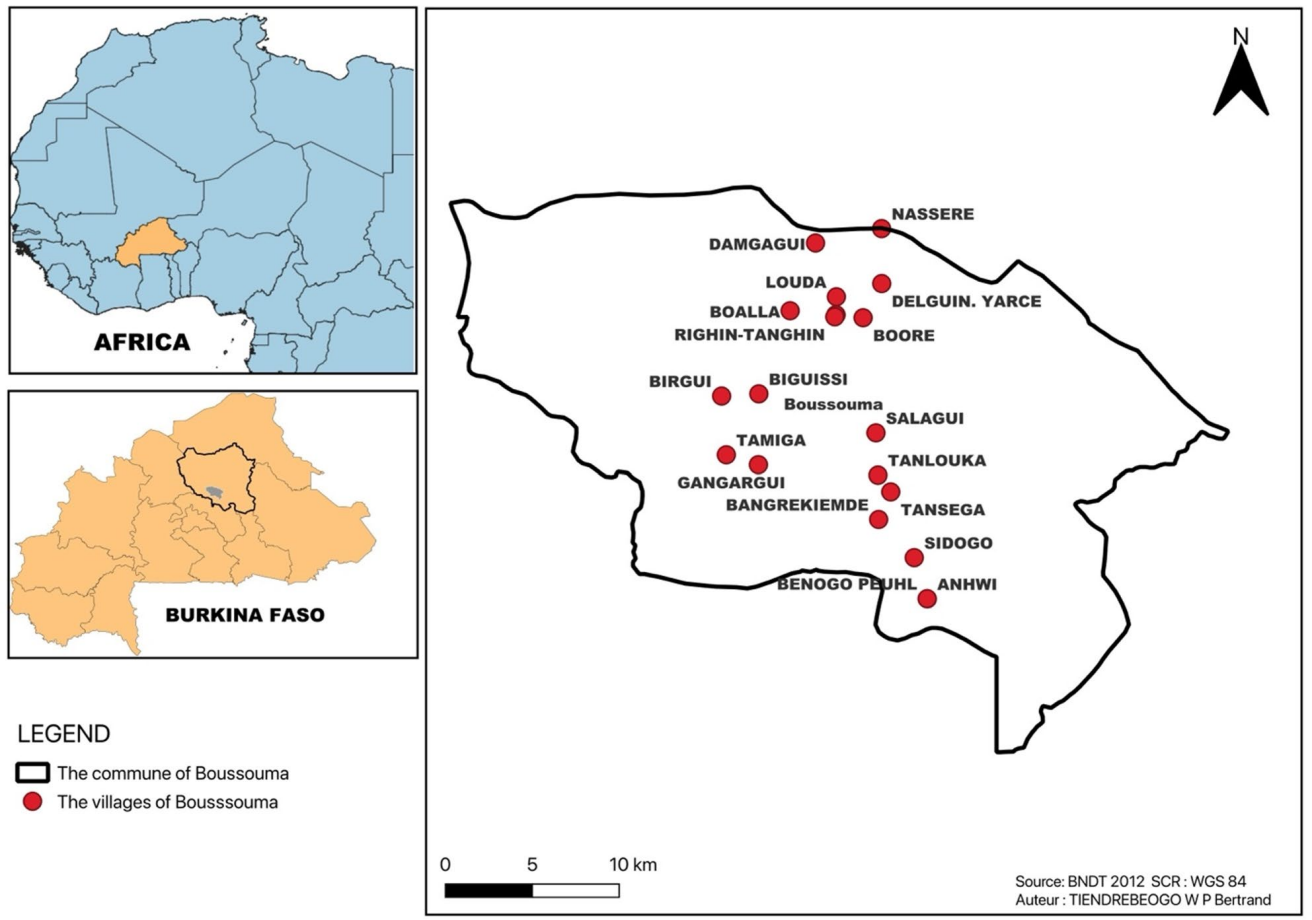
Map of the study area

The study was conducted in the rural commune of Boussouma, located in the province of Sandbondtenga in the Kuilsé region (formerly the North-Central region) of Burkina Faso. The commune covers approximately 770 km² and includes 63 administrative villages. According to the Municipal Development Plan (PCD, 2020), the population was estimated at 131,133 inhabitants in 2024, distributed across 19,536 households, of whom 60,605 were men (46.2%) and 70,527 were women (53.8%) [14] (Fig. 1).

### Sampling plan and household selection

This cross-sectional study was conducted within the framework of the Poultry Losses and One Health (POLOH) project. Due to the unstable security context in the area, purposive sampling was used with the support of local authorities to select accessible and secure villages. Of the 62 villages in the commune, 23 were included based on these criteria. A preliminary census identified 483 households engaged in traditional poultry farming within the selected villages. From this list, 73 households were randomly selected for microbiological sampling using a computer-generated randomization procedure implemented in Microsoft Excel. Additional households were randomly selected as replacements in each village in case of non-participation or exclusion. The inclusion criteria required households to practice traditional chicken farming and not administer antibiotics to their birds during the two weeks preceding sampling.

### Sample size calculation

#### Risk factor analysis

A three-stage procedure was used to compute the sample size in the study. The sample size calculation considered exposure prevalence, household clustering, and statistical power. The standard formula for comparing two proportions was used to calculate the initial sample size:

$$n=\frac{{{(Z}_{(1-\alpha/2)}+{Z}_{1-\beta})}^{2}\times[{P}_{1}\left(1-{P}_{1}\right)+{P}_{2}\left(1-{P}_{2}\right)]}{{({P}_{1}-{P}_{2})}^{2}}$$

Where n is the required sample size per group.

${Z}_{(1-\alpha/2)}$ = is the normal deviate that provides 95% confidence interval (the value of Z is 1.96),

${Z}_{1-\beta}$ = standard normal deviate corresponding to the desired statistical power (0.84 for 80% power) ;

${P}_{1}$= expected prevalence in the exposed group ;

${P}_{2}$ = expected prevalence in the unexposed group.

Based on the estimated Salmonella prevalence of 60% in exposed households and 40% in unexposed households, the required sample size was 95 chickens per group.

### Adjustment for clustering

A Design Effect (DE**)** was applied to correct for the lack of independence between chickens in the same household (since 4 chickens were sampled concurrently using the formula :

$DE=1+(m-1)\times ICC$ (Number of chickens sample per household (m = 4) ; Intra Correlation Coefficient = 0.06).

### Accounting for exposure

In order to guarantee that the “exposed” group (those with improper waste disposal) had the required no of chickens, the total sample was inflated, given that only 40% of households were expected to demonstrate the exposure. This raised the estimate to 290 chickens from 73 households.

### Animal-level prevalence

The required sample size was calculated assuming an expected *Salmonella* spp. prevalence of 53% based on previous studies [11], a desired precision of 7%, and a 95% confidence level, yielding a minimum of 196 samples without clustering. Considering cluster sampling at the household level (for birds per household) and intra-class correlation (ICC) = 0.06 [15], the adjusted sample size increased to 232 samples (58 households).

To satisfy the requirements for both prevalence estimation and risk factor analysis, the largest calculated sample size was retained. Consequently, a total of 292 chickens (four per household) from 73 households were included in the study.

### Selection and collection of poultry samples

In each household, four adult indigenous chickens aged 3–12 months were selected, yielding a total of 292 samples. All sampled birds were privately owned and raised under traditional free-range production systems in the rural commune of Boussouma.

Data collection was conducted in two stages. In the first stage, a structured household-level questionnaire was administered to collect information on poultry management practices, hygiene conditions, biosecurity measures, and household demographics. The questionnaire was developed based on a review of the literature and field experience in traditional village poultry systems [16, 17]. It was pre-tested in five households to ensure clarity and reliability, translated into the local languages (Mooré), and administered by the same trained investigators to ensure consistency. A second visit was conducted within 3–5 days after the initial survey to ensure consistency and minimize recall bias. During this visit, individual chicken-level information including age, treatment history, and vaccination status was recorded prior to biological sample collection. In this study, a caregiver was defined as any household member, other than the household head, who was regularly involved in poultry-related activities such as feeding, cleaning, watering, or monitoring the birds. An English version of the questionnaire is provided as Supplementary Material (Additional file 1).

Birds were identified using observable physical characteristics (e.g., feather color, size, and comb type), and random selection was performed using a simple randomization procedure. In households with more than 50 chickens, birds were first grouped by age or housing area prior to random selection to ensure representation across flock subgroups.

Chickens were humanely euthanized on site by cervical dislocation, performed by trained personnel in accordance with the American Veterinary Medical Association (AVMA) Guidelines for the Euthanasia of Animals [18]. Cecal intestinal contents were aseptically collected using sterile gloves, placed in labeled sterile bags, and stored in coolers at 4 °C until further processing.

### Transport and storage conditions

Samples were transported within four hours in temperature-controlled cooler boxes to the Laboratory of Molecular Biology, Epidemiology, and Surveillance of Foodborne Bacteria and Viruses (LaBESTA) at Joseph Ki-Zerbo University. Laboratory processing began immediately upon arrival.

### Microbiological analyses

#### Detection of *Salmonella* spp.

*Salmonella* spp. detection followed the ISO 6579:2012 standard. One gram of cecal content was pre-enriched in 9 mL of sterile Buffered Peptone Water (BPW) at 37 °C for 24 h. For selective enrichment, 100 µL of BPW culture was transferred into 9.9 mL of Rappaport–Vassiliadis (RV) broth and incubated at 42 °C for 24 h. Isolation was carried out by streaking 10 µL from each RV tube onto *Salmonella* spp. Chromogenic Agar plates at 37 °C for 24 h. Presumptive colonies (add what they would look like) were biochemically confirmed using Kligler-Hajna iron agar, Triple Sugar Iron, urease, and indole. Confirmed isolates were stored at − 80 °C in cryovials with 15% glycerol.

### MALDI-TOF MS confirmation

All confirmed isolates were subjected to species-level identification using the Bruker Biotyper (Bruker Daltonik GmbH), which employs matrix-assisted laser desorption/ionization time-of-flight mass spectrometry (MALDI-TOF MS). The direct spotting method was performed according to the manufacturer’s instructions.

#### Quantification of *Salmonella* spp.

For *Salmonella* quantification, one gram of cecal content was homogenized in 9 mL of buffered peptone water (BPW) by vortexing. Following homogenization, serial ten-fold dilutions (10⁻² and 10⁻³) were prepared in BPW. All BPW aliquots were subjected to pre-enrichment at 37 °C for 18 h. Subsequently, 0.1 mL from each pre-enrichment tube was transferred into 9.9 mL of Rappaport–Vassiliadis broth and incubated at 42 °C for 24 h. After selective enrichment, aliquots were streaked onto *Salmonellla* Chromogenic agar plates and incubated at 37 °C for 24 h. Presumptive Salmonella colonies were selected and confirmed using standard biochemical tests. The number of Salmonella-positive tubes at each dilution level was then used to estimate bacterial concentrations, expressed as Most Probable Number per gram of fecal sample (MPN/g) [19].

### Data analysis

#### Prevalence estimation

The animal level prevalence was calculated as the proportion of positive samples among the total samples examined. Flock-level prevalence was defined as households. Exact 95% confidence intervals (CI) using the Clopper–Pearson method.

#### Risk factor analysis

Data were analyzed using Stata version 14 (StataCorp, College Station, TX, USA). Descriptive statistics were first used to summarize household- and animal-level variables.

After univariate analysis, a logistic regression model was fitted to assess associations between potential risk factors and *Salmonella* spp. positivity (binary outcome: presence/absence). Potential risk factors were first screened for association with Salmonella spp. positivity using univariate logistic regression. Independent variables were retained for multivariable analysis when they satisfied a significance level of *p* ≤ 0.20. In addition, variables that did not meet this statistical threshold but were considered biologically plausible and contextually relevant based on field observations and previous studies [20–22] were also retained.

A multivariable logistic regression model was then fitted to identify factors independently associated with *Salmonella* spp. presence. A stepwise selection procedure was applied to retain variables in the final model, with statistical significance set at *p* < 0.05. An OR > 1 indicated increased odds of *Salmonella* spp. detection, while an OR < 1 indicated a protective association. Multicollinearity among explanatory variables was assessed using the Variance Inflation Factor (VIF), and variables with VIF values greater than 5 were excluded from the final model.

#### Assessment of factors that affect *Salmonella* spp. bacterial load in chicken faeces

A linear regression analysis was conducted to examine factors affecting *Salmonella* spp. bacterial load. The model was developed to estimate the average effect of each predictor on bacterial concentration. Forest plots, created in RStudio (version 2025.05.1, Foundation for Statistical Computing, Vienna, Austria), to visualize regression coefficients and 95% pension intervals to facilitate interpretation of the direction and strength of the associations.

The selected explanatory variables were derived from literature research, biological feasibility, and pertinent factors related to traditional poultry farming systems in rural Burkina Faso. Specifically, variables for sociodemographic conditions, husbandry and feeding practice, herd composition, and hygiene and biosecurity measures were included. These groups were selected as they account for the main domains by which *Salmonella* spp. can pass and persist at the household level while incorporating both animal-related and environmental exposure observed during the study.

## Results

### Household sociodemographic characteristics

Table 1 displays the sociodemographic data of the 73 surveyed households. Most respondents were men (94.5%), reflecting the male-dominated structure of household headship in the study area. More than half were aged > 50 years (54.8%). The majority of respondents reported no formal education (78.1%). Most households were headed by married individuals, either monogamous (50.7%) or polygamous (42.5%). Marital status was included as a proxy indicator of household structure and organization, which may influence poultry management through differences in household size, division of labor, caregiving responsibilities, and hygiene practices. Approximately half of the households (50.7%) reported more than 20 years of experience in poultry keeping. Children aged < 5 years were present in 47.9% of households, and among these, 50.7% reporting having no additional caregiver responsible for management besides the primary household head.

**Table 1:** Sociodemographic profile of households involved in backyard poultry farming in Boussouma (*n* = 73)

Variable	Category	*N* (households)	%
Gender	Female	4	5.5
	Male	69	94.5
Age of household head	< 30 years	0	0.0
	30–39 years	14	19.2
	40–49 years	19	26.0
	≥ 50 years	40	54.8
Education level	Educated	16	21.9
	No formal education	57	78.1
Marital status	Married (monogamous)	37	50.7
	Married (polygamous)	31	42.5
	Not married	1	1.4
	Widow(er)	4	5.5
Children under 5	0–1	38	52.1
	2–3	21	28.8
	≥4	14	19.2
Years of experience	< 10 years	16	21.9
	10–19 years	20	27.4
	≥ 20 years	37	50.7
Caregivers	None	37	50.7
	One	23	31.5
	Two or more	13	17.8

### Livestock and farming systems

Most households maintained small poultry flocks, with 50.7% keeping 10–29 chickens and 16.4% keeping fewer than 10 birds (Table 2). Mixed livestock keeping was common: 90.4% of households owned sheep, 57.5% owned goats, 41.1% owned cattle, and 42.5% kept at least one donkey. Guineafowl were present in 23.3% of households, whereas pigeons (4.1%) and ducks (in 1.4%) were rarely kept.

**Table 2:** Distribution of livestock holdings at household level (*n* = 73)

Species	Category	*N* (households)	%
Poultry	Very small (3–9)	12	16.4
	Small (10–29)	37	50.7
	Medium (30–69)	19	26.0
	Large (70+)	5	6.9
Sheep	None	7	9.6
	Small (1–9)	45	61.6
	Medium (10–19)	15	20.5
	Large (20+)	6	8.2
Goats	None	31	42.5
	Small (1–9)	33	45.2
	Medium (10–19)	7	9.6
	Large (20+)	2	2.7
Cattle	None	43	58.9
	1–2	24	32.9
	> 2	6	8.2
Donkeys	None	42	57.5
	1 donkey	21	28.8
	> 1 donkey	10	13.7
Guinea fowl	None	56	76.7
	> 0	17	23.3
Pigeons	None	70	95.9
	> 0	3	4.1
Ducks	None	72	98.6
	> 0	1	1.4

### Characteristics of sampled chickens

All 292 sampled birds were of local indigenous breeds (Table 3). More than half of the birds were aged > 5 months (51.4%), while 34.9% were between 4 and 4.9 months and 13.7% were 3-3.9 months old. The majority of sampled birds were male (72.3%), with females accounting for 27.7% of the sample.

**Table 3:** Demographic characteristics of chickens sampled (*n* = 292)

Characteristic	Category	*N* = 292	%
Age (months)	3–3.9	40	13.7
	4–4.9	102	34.9
	≥ 5	150	51.4
Sex	Male	211	72.3
	Female	81	27.7

### *Salmonell*a spp. prevalence

#### Animal-level prevalence

Of the 292 chickens sampled, 167 were positive for *Salmonella* spp. yielding an animal- level prevalence of 57.2% (95% CI: 51.5–62.9), while 125 samples (42.8%) were negative.

#### Flock-level prevalence

At the household level, 63 of the 73 households had at least one *Salmonella* spp. positive chicken, corresponding to a flock-level prevalence of 91.8% (95% CI: 84.1–97.1). Only six households (8.2%) were classified as negative.

#### Bacterial load distribution of *Salmonella* spp.

Among the 292 chickens sampled, 125 (42.8%) were negative for Salmonella spp. Among the 167 positive chickens, most exhibited low bacterial loads. Specifically, 133 birds (45.5% of all chickens; 79.6% of positives) had low loads (0.0001 to 1 MPN/g), 23 birds (7.9% of all chickens; 13.8% of positives) had moderate loads (1.0001-5 MPN/g), and 11 birds (3.8% of all chickens; 6.6% of positives) had high loads (> 5 MPN/g).

### Risk factors associated with *Salmonella* spp. presence at the animal level

#### Univariate logistic regression

In the univariate logistic regression, several household management and hygiene practices were significantly associated with the presence of *Salmonella* spp. in chickens (Table 4). Poultry treatment was protective (OR = 0.26, 95% CI: 0.12–0.53, *p* < 0.001). In contrast, leaving carcasses on household premises increased the OR of infection (OR = 5.85; 95% CI: 1.70–20.08, *p* = 0.005). The presence of guinea fowl was also associated with higher odds of *Salmonella* spp. detection (OR = 1.78, 95% CI: 1.00–3.15, *p* = 0.048).

Hygiene-related practices were consistently protective. Occasional faeces cleaning (OR = 0.20, 95% CI: 0.06–0.65, *p* = 0.008), frequent cleaning (OR = 0.26, 95% CI: 0.09–0.78, *p* = 0.017) and manure disposal (OR = 0.54, 95% CI: 0.33–0.89, *p* = 0.017) were all associated with reduced *Salmonella* spp. presence.

Sociodemographic factors showed limited associations. Households with two or four children under five years of age had lower odds of infection, while the number of caregivers in the household showed only borderline associations. No significant associations were observed for sex of household head, education level, water source, or night-time confinement of poultry in univariate analysis.

**Table 4:** Results of univariate analyses of factors associated with *Salmonella* spp. presence in chicken

Variable	Reference category	Comparison category	OR	95% CI	*p*-value
Gender	Female	Male	0.43	[0.13–1.36]	0.149
Education level	Educated	No formal education	0.89	[0.51–1.57]	0.690
Number of children < 5 years	0	1	0.61	[0.29–1.27]	0.185
		2	0.36	[0.16–0.80]	0.013*
		3	1.00	[0.34–2.94]	1.000
		4	0.35	[0.13–0.96]	0.042*
Tertiary activity	Agriculture	Gold mining	0.14	[0.01–1.76]	0.129
		Other livestock	1.33	[0.28–6.44]	0.720
Poultry treatment	No	Yes	0.26	[0.12–0.53]	< 0.001**
Vaccination (New Castle )	No	Yes	0.94	[0.58–1.53]	0.815
Sheep herd size	None	Small (1–9)	1.62	[0.73–3.59]	0.240
Goat herd size	None	Small (1–9)	1.70	[1.03–2.80]	0.039*
Cattle herd size	None	1–2	1.48	[0.89–2.47]	0.133
Donkey ownership	None	1 donkey	1.40	[0.80–2.35]	0.243
		> 1 donkey	1.27	[0.63–2.56]	0.500
Presence of guinea fowls	None	≥ 1	1.78	[1.00–3.15]	0.048*
Night confinement of poultry	No	Yes	1.30	[0.79–2.14]	0.303
Carcass burial	No	Yes	1.30	[0.82–2.07]	0.272
Manure disposal	No	Yes	0.54	[0.33–0.89]	0.017*
Faeces cleaning	Never	Occasional	0.20	[0.06–0.65]	0.008**
		Frequent	0.26	[0.09–0.78]	0.017*
Abandonment of carcasses	No	Yes	5.85	[1.70–20.08]	0.005**
Water supply	No	Yes	1.08	[0.68–1.73]	0.742
Number of caregivers	None	One	1.62	[0.94–2.79]	0.083
		Two or more	0.57	[0.30–1.09]	0.089

*OR *Odds Ratio, *CI *Confidence Interval

Significance levels : * *p* < 0,05 ; ** *p* < 0,01 ; *** *p* < 0,001

#### Multivariate logistic regression model

In the final multivariate logistic regression model, lack of poultry treatment remained strongly associated with *Salmonella* spp. presence (OR = 3.77; 95% CI: 1.70–8.32; *P* = 0.001). Leaving poultry carcasses on household premises was also a major independent risk factor (OR = 5.61; 95% CI: 1.48–21.23; *P* = 0.011), as was ownership of more than one donkey(OR = 4.30; 95% CI: 1.63–11.35; *P* = 0.003). Protective factors included access to improved water sources (OR = 0.46; 95% CI: 0.24–0.88; *P* = 0.020) and manure disposal (OR = 0.44; 95% CI: 0.23–0.85; *P* = 0,013). Each additional child under five years of age was weakly associated with reduced odds of *Salmonella* spp. detection (OR = 0.89; 95% CI: 0.81–0.98; *P* = 0.017). Male-headed households are significantly less likely to be contaminated (OR = 0.22; 95% CI: 0.06–0.86; *P* = 0.029). Second, night-time confinement of poultry shows a marginal association with an increased risk, although this trend does not reach the conventional threshold for statistical significance (*p* = 0.051).

**Table 5:** Logistic regression model of risk factors associated with *Salmonella* spp. presence in households in Boussouma

Variable	OR	Std. Error	95% CI	*P*-value
Poultry treatment	3.77	1.52	[1.70–8.32]	0.001**
Tertiary activity	1.45	0.14	[1.21–1.75]	0.001**
Carcass abandonment on-site	5.61	3.81	[1.48–21.23]	0.011*
Manure disposal	0.44	0.15	[0.23–0.85]	0.013*
Water source	0.46	0.15	[0.24–0.88]	0.020*
Number of children < 5 years	0.89	0.04	[0.81–0.98]	0.017*
Donkey herd (> 1)	4.30	2.13	[1.63–11.35]	0.003**
Carcass burial	1.92	0.56	[1.09–3.39]	0.024*
Sex (male)	0.22	0.15	[0.06–0.86]	0.029*
Night-time poultry confinement	1.86	0.59	[1.00–3.46]	0.051
Constant	0.17	0.16	[0.03–1.06]	0.058

*OR *Adjusted Odds Ratio, *Std. Error* Standard Error of the OR, *CI* Confidence Interval

Significance levels : * *p* < 0,05 ; ** *p* < 0,01 ; *** *p* < 0,001

#### Factors associated with *Salmonella* spp. bacterial load: linear regression analysis

**Fig. 2:**
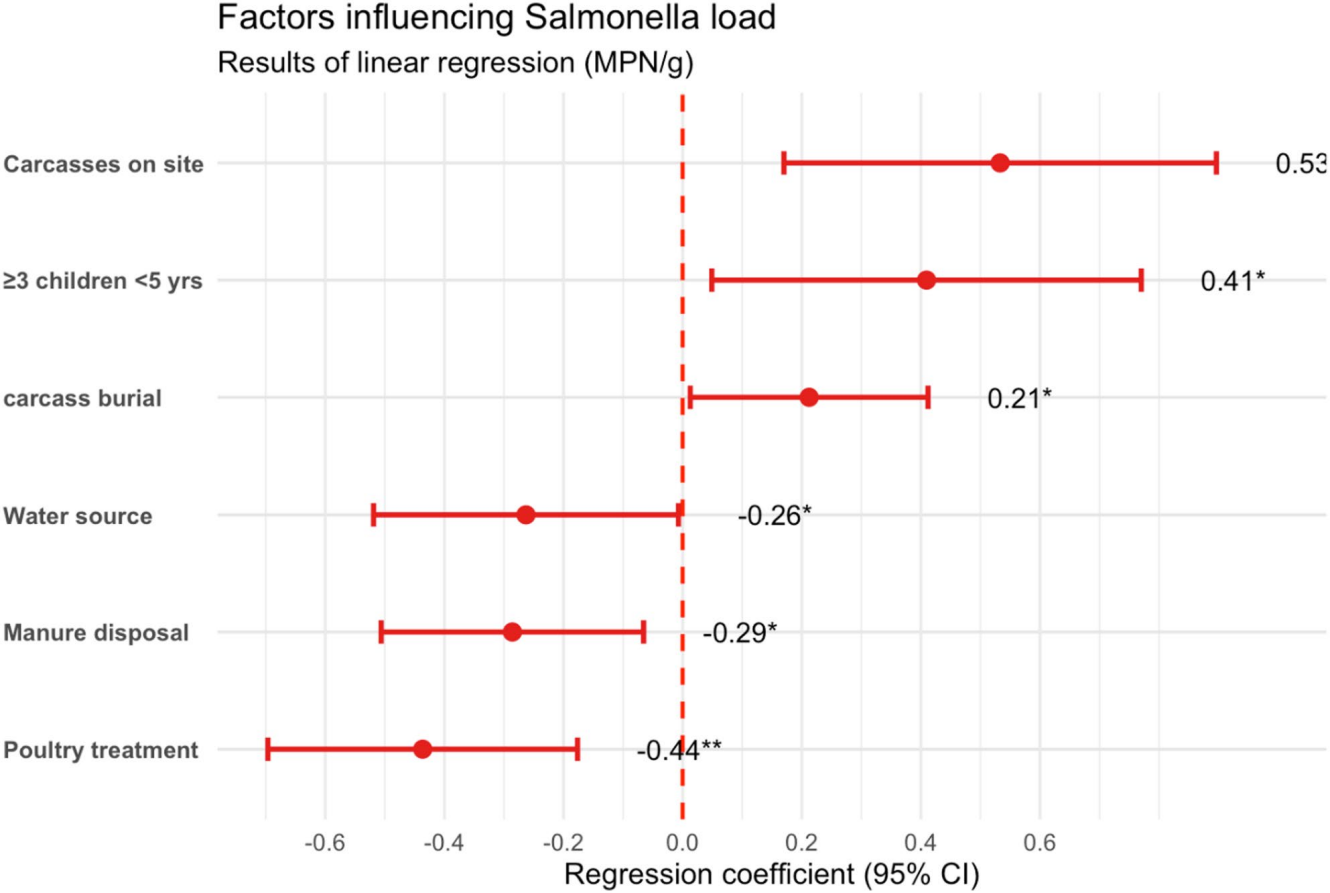
Forest plot of linear regression coefficients for the quantitative load of *Salmonella* spp. (MPN/g). Points show estimated coefficients with 95% confidence intervals. The dashed line indicates the null effect (coefficient = 0). Positive values indicate increased load and negative values indicate decreased load. Red points indicate statistically significant associations (* *p* < 0.05; ** *p* < 0.01; *** *p* < 0.001)

A multiple linear regression is performed to identify factors associated with the quantity of *Salmonella* spp., expressed in MPN/g. The model was statistically significant (F (25,266) = 3.88; *p* < 0.001) and explains approximately 20% of the observed variation (adjusted R² = 0.198). Our results show that certain factors significantly influence *Salmonella* spp. load. Specifically, leaving poultry carcasses on site is associated with a mean increase of 0.53 log MPN/g, while burying carcasses is linked to a mean increase of 0.21 log MPN/g. In contrast, access to a borehole water supply reduces the load by approximately 0.26 log MPN/g, and manure disposal is associated with a mean reduction of 0.29 log MPN/g. The administration of treatments also shows efficacy, with an estimated reduction of 0.44 log MPN/g. Finally, the presence of three children under five years of age in the household is associated with an increase in the mean bacterial load of 0.41 log MPN/g (Fig. 2).

## Discussion

### Animal and flock levels prevalence of *Salmonella* spp.

At the animal level, *Salmonella* spp. was detected in 57.2% (95% CI: 51.5–62.9) of chicken fecal samples in Boussouma, revealing a widespread contamination among village poultry flocks. This prevalence is considerably higher than that reported in urban poultry settings in Ouagadougou [23], where *Salmonella* spp. were found in 9.9% of chicken droppings, and in the Mekong Delta (Vietnam) by Nguyen [24], where fecal samples showed a prevalence of 7.7%. However, it is close to the 40.8% observed in free-ranging chickens in Ethiopia [25]. These variations in production systems, hygiene practices, and environmental exposure, particularly the free-scavenging nature of village poultry farming in rural Burkina Faso, likely explain this high prevalence as similarly reported in other low-input poultry systems in sub-Saharan Africa [5]. Despite this widespread occurrence, most positive chickens carried only low bacterial loads. In fact, 45.6% of sampled birds harbored minimal amounts of the pathogen (0.0001–1 MPN/g), whereas moderate and high loads were observed in only 7.9% and 3.8% of birds, respectively. This distribution indicates that the majority of infected birds serve as asymptomatic or persistent carriers rather than suffering from acute clinical disease [5] which is increasingly recognized as a key driver of environmental persistence of *Salmonella* spp [4].

In traditional poultry systems such as those in Boussouma, repeated exposure to contaminated soil, manure, and household environments may encourage persistent low-level colonization and continuous environmental recycling of *Salmonella* spp. This silent circulation represents a significant qualitative risk, as it allows long-term contamination of household spaces without producing obvious signs of illness in the poultry [5].

At the flock level, *Salmonella* spp. was detected in 91.8% (95% CI: 84.1–97.1) of surveyed households, indicating that contamination is nearly ubiquitous in traditional poultry settings. The presence of multiple animal species and the lack of routine cleaning, disinfection, and litter management likely increase environmental persistence and cross-contamination between flocks. Under such conditions, even low bacterial loads at the individual animal level may cumulatively sustain widespread household contamination.

These findings indicate that *Salmonella* spp. is deeply entrenched in household poultry systems in rural Burkina Faso, not primarily through severe disease in birds, but through widespread low-level carriage and persistent environmental cycling.

### Risk factors for *Salmonella* spp. infection in a chicken

This study identified several significant risk factors for *Salmonella* spp. detection in rural poultry systems reflecting practical vulnerabilities in household-level management and hygiene practices at the animal–household interface.

The absence of veterinary treatment was strongly associated with *Salmonella* spp. presence (OR = 3.77; 95% CI: [1.70–8.32]; *p* = 0.001). In Boussouma, limited access to veterinary services, poor road infrastructure, and financial constraints often lead farmers to rely on informal self-medication using antibiotics purchased from local markets. While such practices may reduce *Salmonella* spp. prevalence or bacterial loads in the short term, they also raise concerns regarding inappropriate antimicrobial use and the potential selection of antimicrobial-resistant strains.

Similar patterns were reported in Kenya and Ethiopia [26, 27], and the FAO notes that weak veterinary coverage in low-resource settings fosters pathogen persistence [28].

Carcass management was another key determinant. Households that abandoned dead birds on-site (OR = 5.61; 95% CI: [1.48–21.23]; *p* = 0.011) or buried them near living areas (OR = 1.92; 95% CI: [1.09–3.39]; *p* = 0.024) faced elevated risks. These practices likely enhance environmental contamination and facilitate indirect transmission, particularly in household courtyards where poultry, children, and other animals interact closely. Comparable findings were reported in Nigeria and Ethiopia [20, 29].

On the other hand, households that properly disposed of poultry manure (OR = 0.44; 95% CI: [0.23–0.85]; *p* = 0.013) or used improved water sources (OR = 0.46; 95% CI: [0.24–0.88]; *p* = 0.020) showed significantly lower prevalence. These practices reduce fecal-oral transmission and limit the environmental survival of *Salmonella* spp., consistent with findings from other rural poultry systems [13, 30].

Sociodemographic factors also influenced infection risk. Households with a higher number of children under five years of age were less likely to show *Salmonella* spp. presence (OR = 0.89; 95% CI: 0.81–0.98; *p* = 0.017). This finding may reflect greater vigilance and improved hygiene practices when young children are present. In contrast, when bacterial load was considered, households with three or more children under five exhibited higher *Salmonella* spp. concentrations, which suggests increased environmental contamination through frequent contact with poultry, soil, and faeces. This apparent discrepancy highlights the complexity of exposure pathways. Although heightened awareness may help reduce the risk of infection, persistent low-level exposures can perpetuate high bacterial burdens after contamination has occurred.

Households headed by men (OR = 0.22; 95% CI: [0.06–0.86]; *p* = 0.029) were less affected. These results may reflect gendered disparities in access to sanitation and veterinary services [31–33].

Economic activity also played a role. Poultry, often perceived as a marginal activity, was the.

third income source reported by households, and was termed “tertiary activity”. “Tertiary activity” in the study site was dominated by poultry farming (OR = 1.45; 95% CI: [1.21–1.75]; *p* = 0.001). Though considered minor, poultry provides a flexible source of cash and protein and is often managed with minimal supervision. This marginal status limits investment in hygiene and veterinary care, thereby increasing the risk of contamination [34, 35].

Finally, mixed livestock rearing, particularly the presence of more than one donkey (OR = 4.30; 95% CI: [1.63–11.35]; *p* = 0.003), was associated with a higher risk. The lack of species segregation can facilitate cross-contamination via shared soil and water sources, as observed in Rwanda and Nigeria [20, 36].

These findings underscore the importance of implementing targeted interventions, including expanding veterinary outreach, promoting hygiene education, and enhancing waste management, to mitigate the transmission of *Salmonella* spp. and safeguard public health in rural Burkina Faso. One Health-oriented *Salmonella* spp. surveillance and hygiene-focused interventions along the poultry value chain need to be integrated in the control of *Salmonella* spp.

### Factors that affect *Salmonella* spp. bacterial load in chicken faeces

The linear regression model identified six significant factors influencing the bacterial load of *Salmonella* spp. in chicken faeces. These determinants reflect hygiene practices, access to basic services, and household dynamics, with direct implications for poultry production and public health. Similar observations have been reported in rural Burkina Faso, where poultry husbandry and household hygiene practices were associated with both poultry health and child nutrition outcomes [37, 38].

Abandonment of poultry carcasses on-site (Coef. = 0.53; *p* = 0.004) was strongly associated with increased bacterial load. Leaving dead birds exposed facilitates environmental contamination and bacterial proliferation, especially in courtyards shared with children and other animals. Previous studies have demonstrated that *Salmonella* spp. concentrations in poultry litter can range from 0.45 to over 280,000 MPN/g, confirming that fecal contamination levels vary widely depending on management practices [39]. Similar risks were reported by Miller [40], who found that poor carcass disposal contributes to pathogen persistence in rural environments.

Similarly, carcass burial (Coef. = 0.21; *p* = 0.037) also contributed to higher contamination levels. Although intended as a hygienic measure, shallow or poorly located burials may lead to recontamination of soil and water. Gumasta [41] highlighted that burial near living areas increases bacterial load, especially when carcasses are not properly covered or isolated.

In contrast, access to improved water sources (Coef. = − 0.26; *p* = 0.044) and proper manure disposal (Coef. = − 0.29; *p* = 0.011) were associated with significantly lower bacterial loads. These practices reduce fecal-oral transmission and limit environmental persistence of *Salmonella spp.*, consistent with findings from previous studies [30] in Tanzanian poultry systems. Clean water reduces the risk of recontaminating feed and drinking points, while safe manure handling prevents pathogen spread to soil and nearby dwellings.

Veterinary treatment (Coef. = − 0.44; *p* = 0.001) was another protective factor. Households that administered antibiotics had significantly lower bacterial loads, underscoring the importance of veterinary access. Asfaw [27] and the FAO [28] emphasize that veterinary oversight reduces pathogen circulation and improves poultry health outcomes.

Finally, the presence of three children under five years old (Coef. = 0.41; *p* = 0.026) was linked to higher bacterial load. In such households, hygiene management may be more challenging due to competing childcare responsibilities. Close contact between children and domestic animals has been associated with increased exposure to enteric pathogens [33, 42], which may indirectly contribute to environmental contamination.

These findings highlight the need for targeted interventions by improving carcass and manure management, expanding access to clean water and veterinary services, and supporting hygiene education in households with young children. Reducing bacterial load in poultry faeces is essential not only for flock health but also for limiting zoonotic transmission in rural communities.

### Recommendations

This study underscores the importance of practical, household-level measures to reduce *Salmonella* spp. contamination in village poultry systems in rural Burkina Faso. Simple, low-cost hygiene practices such as safe manure disposal, wet cleaning rather than dry sweeping, and basic biosecurity should be prioritized, even though poultry often represents a tertiary income source with limited labor and financial investment. While access to improved water sources was linked to lower *Salmonella* spp. prevalence, high costs, and limited availability remain significant barriers. In the meantime, promoting safer water handling and reducing reliance on contaminated surface water can provide realistic alternatives. Because poultry treatment practices were associated with *Salmonella* spp. detection, interventions must discourage unsupervised antibiotic use and strengthen veterinary support to limit both infection and antimicrobial resistance. Finally, households with young children should be a key focus for hygiene education, as they face greater exposure once contamination occurs. Together, these context-adapted, household-centered strategies are essential to mitigating zoonotic risks.

### Limitations of the study

This study was conducted in a single rural commune, which may limit the generalizability of the findings to other settings. Sampling was performed at a single time point; therefore, potential seasonal variations in *Salmonella* spp. prevalence and bacterial load could not be assessed. Because chickens were sampled within households, the results may reflect clustering effects related to shared environments and management practices. Village selection was guided by accessibility and security considerations, which may have introduced selection bias by excluding more remote or less secure areas.

The cross-sectional design limits causal inference, as observed associations between husbandry practices and *Salmonella* spp. detection cannot establish temporal relationships. In addition, although multivariable models were applied, residual confounding cannot be excluded, as certain factors, such as seasonality, climatic conditions, coinfections, vaccination against other poultry diseases, and pest presence, were not systematically measured.

Finally, the semi-quantitative nature of the Most Probable Number (MPN) method and the absence of serotype-level identification of Salmonella isolates, as MALDI-TOF MS only enabled identification at the genus level, reduces the precision of bacterial load estimates and limits deeper epidemiological characterization of circulating strains.

## Conclusions

This study provides essential baseline evidence on *Salmonella* spp. prevalence, bacterial load, and household-level risk factors in backyard poultry systems in Boussouma, Burkina Faso. Beyond documenting widespread contamination, the findings identify practical entry points for low-cost interventions that can reduce transmission at the animal–household interface. Measures such as improved manure management, safe disposal of dead birds, avoidance of dry sweeping, and simple hygiene practices are feasible even where poultry farming remains a secondary or tertiary livelihood activity.

Community engagement emerges as equally critical. Involving household heads, women, and children can foster the sustained adoption of protective behaviors. Although further longitudinal research and expanded surveillance are needed to clarify seasonal dynamics and causal pathways, this work establishes a strong foundation for targeted veterinary support, hygiene education, and locally adapted prevention strategies. By generating context-specific evidence from an underserved rural setting, the study contributes to integrated approaches that safeguard animal health, reduce household exposure, and strengthen public health resilience.

## Supplementary Information

Supplementary Material 1.

## Data Availability

The data supporting the findings of this research can be obtained from the corresponding author upon reasonable request.

## Abbreviations

CI: Confidence Interval
ICC: Intra-class Correlation Coefficient
MPN: Most Probable Number
OR: Odds Ratio
FAO: Food and Agriculture Organization of the United Nations
spp.: Species (plural form)
Coef.: Regression Coefficient
*p*-value: Probability value
