# Supplementary material for: Prevalence, quantification, and household-level risk factors associated with Salmonella spp. infection in chickens in Boussouma commune, Burkina Faso
Source: BMC Microbiol. 2026 Mar 4;26:333. doi: 10.1186/s12866-026-04895-y (PMC13069701; doi:10.1186/s12866-026-04895-y)
Supplement: Supplementary file 1 — Supplementary Material 1. [file 12866_2026_4895_MOESM1_ESM.docx]

**Supplementary file 1 – Household and poultry management questionnaire**

**A. Household Identification**

- Household ID: ______
- Chicken ID: ______

**B. Socio-demographic characteristics of respondents**

- Gender of household head: Male / Female
- Age of household head (years): ______
- Marital status: Married (monogamous)/Married (polygamous)/ Not married/ Widow(er)
- Level of education: None / Primary / Secondary / Higher
- Primary activity of respondent: ______
- Secondary activity of respondent: ______
- Tertiary activity of respondent: ______
- Years of experience in poultry farming: ______

**C. Household composition**

- Number of boys under 5 years: ______
- Number of girls under 5 years: ______
- Number of caregivers in the household: ______

**D. Poultry and animal health**

- Age of chicken (months): ______
- Sex of chicken: Male / Female
- Health status
- Has the chicken received treatments? Yes / No
- If yes, specify: Antibiotics / Dewormers / Other
- Treatment period (days)
- Vaccinated: Yes / No
- If yes, specify disease: Newcastle/ Fowl pox/ Other(specify)/ I don’t know
- Date of last vaccination

**E. Poultry production**

- Quantity of live chickens produced in the last 12 months: ______
- Poultry flock size: ______

**F. Livestock ownership (Number of animals)**

- Sheep: ______
- Goats: ______
- Cattle: ______
- Donkeys: ______
- Dogs: ______
- Cats: ______
- Pigs: ______
- Chickens: ______
- Guinea fowls: ______
- Ducks: ______
- Doves/Pigeons: ______

**G. Poultry housing and management**

- Night confinement of chickens: Yes / No
- Day confinement of chickens: Yes / No
- Where do chickens spend the night? ______
- Where do chickens spend the day? ______
- If not confined at night, are you considering building a confinement?

**H. Feeding practices**

- Scavenging: Yes / No
- Supplemented scavenging (termites/insects): Yes / No
- Period of supplementation: ______
- Home-grown cereals (maize, sorghum, millet, beans): Yes / No
- Rice: Yes / No
- Residues: Yes / No
- Purchased poultry feed: Yes / No
- Kitchen waste/leftovers: Yes / No

**I. Water source and hygiene**

- Main source of drinking water for household: ______
- Provision of water for poultry: Yes / No
- Quarantine of new flock/birds: Yes / No
- Regular cleaning of poultry house: Yes / No
- Isolation of sick birds: Yes / No
- Disinfection of feeding equipment: Yes / No
- Cleaning of compound: Yes / No

**J. Waste and manure management**

- Disposal of dead animals?
- Manure disposal: Yes / No
- How often do you clean chicken feces in the confinement area?
- How do you clean chicken feces in a confinement area?
- How often do you clean feces within the compound?
- Frequency of cleaning feces within the compound?
- Method of feces disposal: Bury / Burn / Throw in bush / Compost / Use as fertilizer / Leave on site (do nothing)/ pile inside compound/ pile outside compound/ Throw into environment/ Other?

**K. Handwashing facilities and practices**

- When do you clean your hands after handling birds and waste?
- Can you show where household members wash their hands most often?
- Facility available?
- Water available?
- Soap or detergent available ?
